# FAMSA: Fast and accurate multiple sequence alignment of huge protein families

**DOI:** 10.1038/srep33964

**Published:** 2016-09-27

**Authors:** Sebastian Deorowicz, Agnieszka Debudaj-Grabysz, Adam Gudyś

**Affiliations:** 1 Institute of Informatics, Silesian University of Technology, Akademicka 16, 44-100 Gliwice, Poland

## Abstract

Rapid development of modern sequencing platforms has contributed to the unprecedented growth of protein families databases. The abundance of sets containing hundreds of thousands of sequences is a formidable challenge for multiple sequence alignment algorithms. The article introduces FAMSA, a new progressive algorithm designed for fast and accurate alignment of thousands of protein sequences. Its features include the utilization of the longest common subsequence measure for determining pairwise similarities, a novel method of evaluating gap costs, and a new iterative refinement scheme. What matters is that its implementation is highly optimized and parallelized to make the most of modern computer platforms. Thanks to the above, quality indicators, i.e. sum-of-pairs and total-column scores, show FAMSA to be superior to competing algorithms, such as Clustal Omega or MAFFT for datasets exceeding a few thousand sequences. Quality does not compromise on time or memory requirements, which are an order of magnitude lower than those in the existing solutions. For example, a family of 415519 sequences was analyzed in less than two hours and required no more than 8 GB of RAM. FAMSA is available for free at http://sun.aei.polsl.pl/REFRESH/famsa.

Multiple sequence alignment (MSA) is one of the most important analyzes in molecular biology. Most algorithms use progressive heuristics^1^ to solve the MSA problem. The scheme consists of three stages: (I) calculation of a similarity matrix for investigated sequences, (II) a guide tree construction, (III) greedy alignment according to the order given by the tree. Pairwise similarities can be established in various ways. Some algorithms use accurate although time-consuming methods, such as calculating pairwise alignments of the highest probability^2^ or maximum expected accuracy^3^. Others employ approximated, yet faster approaches, e.g. tuple matching^4^,^5^. As the sizes of the protein families to be analyzed continue to increase, the necessity to calculate all pairwise similarities has become a bottleneck for alignment algorithms. Therefore, several attempts have been made to accelerate this stage. Kalign^6^ and Kalign2^7^ employ Wu-Manber^8^ and Muth-Manber^9^ fast string matching algorithms, respectively, for similarity measurements. This allows thousands of sequences to be aligned in a reasonable timespan. The idea was further extended by the authors of the presented research in Kalign-LCS^10^, which introduced the longest common subsequence to Kalign2 for similarity measurement. This improved both the alignment quality and the execution time. Nevertheless, in view of the most recent developments in high throughput sequencing, biologists are required to align protein families containing tens of thousands of members. Progressive algorithms which calculate and store all pairwise similarities could not be applied to the problems of such a size due to excessive time and memory requirements.

PartTree, a divisive sequence clustering algorithm for building a guide tree without calculating all pairwise similarities^11^, was one of the ideas to tackle the problem. With average time complexity of *O*(*k* log *k*) and space complexity of *O*(*k*) (*k* being the number of sequences in the input set), PartTree was successfully adopted by MAFFT 6 package^12^. As a result tens of thousands of sequences could be aligned on a typical desktop computer. A different approach is presented in Clustal Omega^13^. It uses mBed, an algorithm for embedding sequences into a lower-dimensional space^14^, which requires only *O*(*k* log *k*) exact similarity values to approximate other values. Embedding is combined with sequence clustering with the use of *K*-means algorithm, which prevents the storage of the whole similarity matrix, and keeps memory requirements under control. Both MAFFT and Clustal Omega use tuple matching for similarity calculation.

A different approach to large-scale alignments was introduced in UPP^15^. The algorithm creates a set of profile models on the basis of a subset of sequences referred to as a backbone. The backbone is then used in the process of aligning the remaining elements of the input set.

While MAFFT, Clustal Omega, and UPP are computationally applicable for families of even 100 000 proteins, we show that the quality of the results for such problems is often unsatisfactory. This article presents FAMSA, a progressive multiple alignment algorithm, particularly suitable for large sets of sequences. Pairwise similarities are established, by analogy to Kalign-LCS, on the basis of the longest common subsequences (LCS). Unlike MAFFT and Clustal Omega, FAMSA calculates all pairwise similarities. The process is highly efficient due to the multithreaded, bit-parallel LCS algorithm suited for AVX extensions^16^ of modern processors. Employing a memory-saving, single-linkage algorithm^17^ for guide tree construction, reduces first-stage memory requirements to *O*(*k*). A novel, in-place algorithm of profile alignment which prevents memory reallocations during the progressive stage is an important factor contributing to the computational scalability of FAMSA. As a result, FAMSA is the fastest and most memory-efficient alignment software when large protein families are taken into account. FAMSA’s superiority was observed for sets ranging from thousands to half a million of sequences.

FAMSA is not only efficient, but also very accurate thanks to a number of algorithmic features. They include LCS for similarity measurement, MIQS substitution matrix^18^, and a correction of gap penalties inspired by MUSCLE^4^. The penalties are additionally adjusted to the set size, which is a novel technique in alignment software, particularly useful in large sets of sequences. Misalignments during the progressive stage are fixed with a refinement scheme similar to the one included in QuickProbs 2^19^. Consequently, when sets of a few thousand or more sequences are considered, FAMSA is significantly more accurate than any other algorithm. Interestingly enough, the difference increases along with the growing number of sequences. For instance, for sets exceeding 25 000 proteins, FAMSA aligned 25–45% more columns in a correct manner than UPP or the most accurate variants of MAFFT or Clustal Omega. When the largest benchmark family containing 415 519 sequences was investigated, the difference was even more remarkable—FAMSA successfully restored three times more columns than its competitors, at a fraction of the required time and memory.

The scalability of FAMSA was assessed on extHomFam, a new benchmark generated by analogy to HomFam^13^, by enriching Homstrad^20^ with families from Pfam database^21^. It contains 380 sets of sizes ranging from 218 to 415 519 sequences. The abundance of numerous protein families (*k* > 10000) makes extHomFam particularly representative for large-scale alignment problems, which are of crucial importance in the context of recent advances in high throughput sequencing.

## Methods

Like other progressive algorithms, FAMSA consists of four stages:

- Calculation of pairwise similarities,
- Determination of a guide tree,
- Progressive profile merging according to the guide tree order,
- Optional iterative refinement of the final profile.

Detailed descriptions of the algorithm stages together with analyzes of time and space complexities are provided in the following subsections.

### Calculation of pairwise similarities

The length of a longest common subsequence (LCS) is used in order to determine the pairwise similarities of sequences in the input set. The choice was motivated by the promising results of LCS application in previous studies^10^,^22^. Given two sequences, *A* and *B*, the length of an LCS is the maximal number of perfectly matching columns. This can be considered as an estimation of true pairwise alignment. To compensate the effect of LCS length being larger for longer sequences, the value is normalized by the indel distance (the number of single-symbol insertions and deletions necessary to transform one sequence into another). This distance approximates the misalignment cost, i.e. the number of gaps in the alignment, in which only perfect matches are allowed:


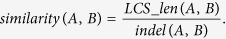


The LCS length can be computed using a straightforward dynamic programming (DP) rule^23^. Owing to the internal properties of the DP matrix, the calculation can be made using the bit-parallel manner. In this approach, *w* cells are computed at a time (*w* is a computer word size equal to 64 in modern architectures)^24^. The indel distance for the sequences *A* and *B* can be directly derived from the LCS length according to the formula:


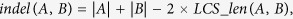


where |*S*| denotes the length of the *S* sequence. The time complexity of the pairwise similarity calculation is:


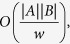


under a reasonable assumption that *w* is comparable to or smaller than the longer sequence length.

As modern computers are equipped with multi-core processors, FAMSA distributes the calculation of LCS lengths for different pairs of sequences to several computing threads. Moreover, the presented software makes use of vector operations provided by technologies like SSE, AVX, AVX 2^16^, which are supported by contemporary processors. This allows multiple pairs of sequences to be processed simultaneously by the same thread. Assuming that there are *t* processing threads, *a* words in a single AVX vector (2 for AVX, 4 for AVX 2, and 8 for the announced AVX-512), and that *n* denotes a sequence length, the total time complexity of the first stage can be expressed as:


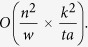


Massively parallel architectures have become widespread in computationally demanding tasks. As FAMSA was designed for analyzing large protein families, it allows massively parallel devices, such as graphics processors, to be employed for the calculation of pairwise similarities. The procedure is implemented in OpenCL^25^, therefore it is suitable for GPUs manufactured by all main vendors, including NVidia and AMD. The distribution of LCS computation over thousands of graphics processor threads further increases the throughput of the first FAMSA stage. Yet, as is shown in the experimental part of the article, even without OpenCL, FAMSA is able to process hundreds of thousands of proteins in a very short time.

### Determination of the guide tree

A number of algorithms for guide tree construction have been developed, e.g. NJ^26^, UPGMA^27^, or single-linkage^28^. FAMSA uses single-linkage for the following reasons:

- it can be computed incrementally, i.e. without storing the complete similarity matrix,
- it is very fast, i.e. it can be completed in *O*(*k*^2^) time using the SLINK algorithm^17^,
- the results were of superior quality in the previous studies^22^,^29^.

To benefit from the incremental property of SLINK, the first two stages of FAMSA are performed simultaneously, which restricts the memory footprint. In particular, tree generation requires only *O*(*k*) space in contrast to *O*(*k*^2^), required by other guide tree construction algorithms like UPGMA. This is of crucial importance when huge protein families are investigated.

### Progressive construction of the alignment

The progressive construction stage requires *O*(*k*) profile alignments, each computed by means of dynamic programming. At least half of these alignments are degenerated cases in which one or both profiles consist of a single sequence. Dynamic programming implementation can be simplified in those cases, hence specialized variants of the general DP procedure were prepared. This resulted in remarkable computation time savings for huge datasets, in which most profile alignments are made against a single sequence due to the structure of a guide tree.

Several improvements in the classical computation rule were made in FAMSA to upgrade the quality of alignment as well as the processing speed. They were possible owing to the internal profile representation composed of three arrays storing:

- occurrence counters of each alphabet symbol in consecutive columns (32n^*^ computer words, with n^*^ being the profile length),
- costs of alignment of consecutive columns to each possible alphabet symbol (also 32n^*^ computer words),
- sequences in the *gapped representation*.

**Figure 1:**
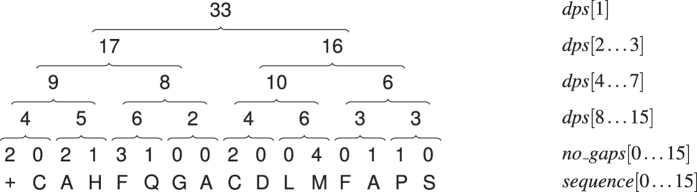
Illustration of gapped sequence representation of – – C A – – H – F – – – Q – G A C – – D L M – – – – F A – P – S. The ‘+’ symbol is a guard, present to simplify the implementation. The values of *dps* are computed according to the rule: *dps*[*i*] = *dps*[2*i*] + *dps*[2*i* + 1], provided that the necessary cells are present. Otherwise, they are calculated on the *no*_*gaps* and *sequence* vectors. For example, *dps*[8] is the number of symbols in *sequence* [0 … 1] (equal to 2), incremented by the number of gaps present just before these symbols, i.e. *no*_*gaps*[0] and *no*_*gaps*[1].

While two former components were previously employed by alignment algorithms, e.g. Kalign, the gapped representation is, to the best of our knowledge, a novel technique. In this representation, two equal-sized arrays are stored for each sequence: (*i*) sequence symbols, (*ii*) the number of gaps present before the corresponding sequence symbol. Moreover, to quickly localize a symbol in a column, as well as to insert or remove gaps, dynamic position statistics are stored in an additional array. The space for the gapped sequence is approximately 13 times the length of the sequence (see Fig. 1 for example). The proposed profile representation allows a dynamic programming matrix to be computed rapidly and is memory frugal. The DP computation step for a pair of profiles takes


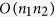


time, where *n*_1_, *n*_2_ are the input profile lengths.

The presence of gapped representation is especially useful when families containing tens of thousands of proteins are investigated. Other aligners construct a new profile by copying the sequences symbol by symbol, with occasional gap insertions, which tends to be a bottleneck for large-scale analyzes. This is not the case in FAMSA, where entire sequences are moved from the input profiles to the new profile and gaps are rapidly inserted by updating gap counters in corresponding arrays. The construction time of a new profile is:


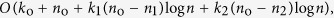


where *k*_1_ and *k*_2_ are the number of sequences in both profiles, *k*_o_ = *k*_1_ + *k*_2_, and *n*_o_ is the resulting profile length. Thus, the overall time of all profile constructions is:


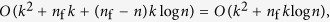


where *n*_f_ is the final profile length.

By adding the time of DP matrix calculation, the total time of this stage is obtained:


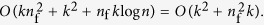


As profile alignments in the bottom part of the guide tree are independent, they can be performed in parallel. Therefore, to improve the computation time, FAMSA distributes profile alignments over multiple threads. It would also be possible to parallelize the dynamic programming computation and construction of a single profile. This is expected to be particularly beneficial for families of a million and more proteins. Nevertheless, this was not done in the current FAMSA version due to implementation complications and unavailability of such large sets in existing databases.

### Determination of gap types and costs

Among numerous amino acid substitution matrices for dynamic programming calculation, MIQS was selected due to superior results reported in the recent study^18^. The gap costs are determined according to the classic affine penalty function, with a distinction between terminal and non-terminal gap open and gap extension costs, by analogy to MUSCLE^4^. In particular, four types of gaps are used:

- terminal gap open (*T*_o_)—opens a sequence at the left end or opens a contiguous series of gaps at the right end of a sequence,
- terminal gap extension (*T*_e_)—extends a series of gaps inserted at the beginning or end of a sequence,
- gap open (*G*_o_)—opens a contiguous series of gaps inserted within a sequence,
- gap extension (*G*_e_)—extends a contiguous series of gaps inserted within a sequence.

While the determination of the number of gaps and their types is straightforward in a pairwise alignment, it becomes problematic in MSA. Due to the fact that before aligning two profiles their sequences may have already contained gaps, the insertion of a column of gaps (either a single one or as the first one in a contiguous series of columns with gaps) does not always mean that exclusively gap opens have been inserted. Inserting only gap opens would result in an overestimation of their number. That is why the types of gaps within a column should be corrected.

**Figure 2:**
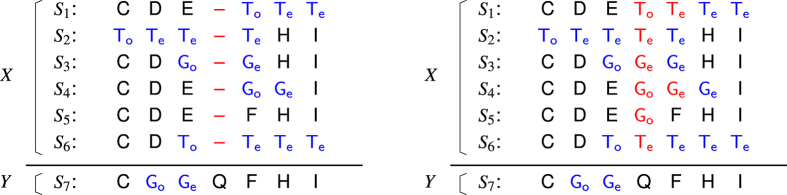
Example of how gap columns are inserted during profile alignment.

Figure 2 demonstrates an example of the alignment of two profiles, *X* and *Y*. A column of gaps is to be inserted into profile *X* (the left part of the figure). The proper types of gaps together with the corrected gaps in the neighboring column are shown in the right part of the figure. The following situations must be considered during correction of the gaps:

- *S*_1_: there is a terminal gap open at the right side of the inserted one; hence, the inserted gap should be a terminal gap open, and the following gap should be transformed into a terminal gap extension,
- *S*_2_: there is a terminal gap extension at the left side of the inserted one; hence, the inserted gap should also be a terminal gap extension,
- *S*_3_: the inserted gap will be placed into the gap series, so it should be a gap extension,
- *S*_4_: there is a gap open at the right side of the inserted one; hence, the inserted gap should be a gap open, and to prevent the occurrence of two gap opens one after the other, the second gap should turn into a gap extension,
- *S*_5_: the inserted gap is to be placed within the series of residues as the only gap, so it should be gap open,
- *S*_6_: there is a terminal gap open at the left side of the inserted one; hence, the inserted gap should be a terminal gap extension.

The optimization of gap parameters and recognition of their influence on alignment accuracy is still the subject of intensive studies^30^. Various techniques have been proposed, e.g. adding a bonus score to a gap cost to enforce the alignment of distantly related sequences^7^. In our research, all gap costs (i.e. gap opens and gap extensions, both terminal and non-terminal) are multiplied by a factor related to the number of sequences in the input collection. This prevents unnecessary widening of alignments of large collections. The scaling factor is calculated as:


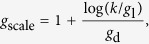


where *g*_l_ and *g*_d_ are two constants set by default to 45 and 7 (values chosen experimentally).

The application of gap corrections and scaling leads to another modification of the traditional approach. It is usually assumed that the insertion of a gap column in to the first profile cannot be immediately followed by the insertion of a gap column in to the second profile. If some assumptions about the gap costs and substitution matrix values are made, it can be proved to be reasonable, i.e. such a situation never leads to the optimal alignment. Nevertheless, this does not hold true if the gap correction is applied. Therefore, it is checked whether consecutive insertions of gap columns in to both profiles render a higher-scored alignment. (The profile alignment score is the summed alignment score of each sequence pair).

### Iterative refinement

The idea of an iterative refinement is to correct misalignments made in the early phase of the profile alignment. Several algorithms were proposed for this task, like REFINER^31^ or the methods implemented in MMSA^22^, MSAProbs^32^. This problem was investigated in our recent paper^19^. It was shown that the classical methods do not work for sufficiently large collections of sequences. A column-oriented refinement was also proposed to improve the quality of alignments for collections up to 1000 sequences. In this approach, the algorithm scans the profile to localize columns that contain at least one gap. Then, it randomly selects one of such columns and splits the profile into two subprofiles, depending on the gap presence in the selected column. Empty columns are removed afterwards, and subprofiles are realigned. Finally, if new alignment is scored higher than the original one, it is accepted as the current solution.

To simplify the time complexity analysis of the refinement, the input and the output profiles are assumed to be of comparable lengths (which is usually the case). A single refinement iteration requires


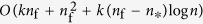


time, with n_*_ being the length of the shorter of the two profiles obtained after splitting the original profile.

Preliminary analyzes showed the refinement to be particularly beneficial for smaller sets of sequences. For this reason, and to improve the processing time of large protein families, the refinement is applied only for *k* ≤ 1000. The number of iterations was experimentally set to 100.

## Results

### Benchmark selection

An assessment of MSA algorithms was performed using benchmark datasets. The presence of high-quality, manually curated reference alignments allowed supervised accuracy measures to be calculated. They were sum-of-pairs (SP) and total-column (TC) scores, defined as fractions of correctly aligned symbol pairs and columns, respectively. The scores were determined with a use of QSCORE software^33^.

Our aim was to propose an efficient and robust algorithm for the alignment of thousands of proteins. The largest available benchmarks contain sets of at most hundreds of sequences, with an exception of HomFam introduced by Sievers *et al*.^13^. HomFam consists of 92 families constructed by extending Homstrad reference alignments (only those having 5 or more sequences were taken into account) with corresponding families from Pfam database. This protocol results in large benchmark sets: 18 of them consist of more than 10 000 members (the number of reference sequences ranges from a few to a few tens, though).

Since 2011, when HomFam was introduced, Pfam and Homstrad databases have demonstrated significant growth. Hence, we present the new benchmark, extHomFam, to carry out more extensive experiments. It was constructed according to the HomFam generation protocol, with several modifications. 399 high-quality alignments containing at least 3 proteins were selected from Homstrad (ver. 1 Apr 2015). By decreasing the threshold from 5, the larger benchmark could be obtained than the original HomFam. If two-protein families were also taken into account, extHomFam would be increased to 1013 sets, at the cost of positively biasing the TC score (with two reference sequences it becomes equal to SP). Therefore, pairwise-only alignments were excluded. Next, selected Homstrad sets were enriched with the corresponding Pfam (ver. 28) families. After removing duplicated sequences, sets of less than 200 proteins were filtered out to provide the final benchmark of 380 families. For convenience extHomFam was divided at thresholds *k* = 4000, 10000, and 25000 to obtain subsets named *small*, *medium*, *large*, and *extra-large*. Note, that sets of ~1000 sequences are usually referred to in the literature as large—our naming convention is intended to show size diversity. *ABC_tran*, the most numerous set in extHomFam, contains 415 519 sequences, which is the largest benchmark protein family available.

The scalability of algorithms was evaluated on the 53 largest extHomFam families, containing at least 30000 sequences each. These sets were recursively downsampled to the desired sizes, with a guarantee of preserving sequences from reference alignments. This scheme has a valuable property: smaller sets are contained in larger ones, which reduces results variability.

To evaluate the performance of the presented algorithm on smaller alignment problems, classic benchmarks, i.e. BAliBASE^34^, PREFAB^4^, OXBench-X^35^, and SABmark^36^, were also considered in the experiments.

### Competitive algorithms and system setup

From among numerous sequence alignment algorithms, only those able to handle families of thousands of sequences were investigated on HomFam and extHomFam. They were MUSCLE^4^, Kalign2^7^, Kalign-LCS^10^, UPP^15^, Clustal Omega^13^ and MAFFT^37^. MAFFT was analyzed in default configuration in which it calculates *O*(*k*^2^) pairwise similarities, as well as -parttree and -dpparttree modes, especially suited for large sets of sequences due to lower computational requirements. Clustal Omega was executed with default parameters and with two combined iterations (-iter2), which delivered superior results in the previous studies^13^,^38^. MUSCLE in default mode was unfeasible for immense protein families, hence -maxiters2 variant was also considered. Details on execution parameters and program versions are given in the Supplementary material.

The experiments on smaller benchmarks (BAliBASE, PREFAB, OXBench-X, SABmark) included also top consistency-based algorithms: MSAProbs^32^, QuickProbs^19^,^39^, and GLProbs^40^.

A workstation equipped with two 12-core Intel Xeon E5-2670v3 processors (clocked at 2.3 GHz), Nvidia Quadro M6000 graphic card (3072 cores clocked at 1.0 GHz), and 128 GB RAM was used for the experiments. To investigate the behavior of the algorithms on modern workstations and servers containing from a few to several tens of cores, all methods were run with 8 computing threads, unless stated otherwise. FAMSA was run in the CPU mode, except for the experiment on the algorithm scalability w.r.t. the number of CPU cores, where the GPU variant was additionally investigated.

### HomFam and extHomFam benchmark evaluation

Following Sievers *et al*.^13^, HomFam was divided into three parts depending on the family size. As is shown in Table 1, for *k* ≤ 3000, FAMSA was inferior only to both Clustal Omega configurations. For *k* > 3000, the presented algorithm took the lead in both measures, revealing its potential for large protein families. More importantly still, FAMSA was from several to hundreds of times faster than its competitors. For instance, it processed the entire HomFam in less than 12 minutes while Clustal-default and MAFFT-default required, respectively, 8 h 40 m and 2 h 30 m. An even greater difference was observed for Clustal-iter2, which completed the analyzes in over 51 hours. Interestingly enough, MAFFT-parttree and -dpparttree were also inferior to FAMSA. This is especially noteworthy because they calculate only selected pairwise similarities (usually *O*(*k*log *k*)) instead of the full matrix (*O*(*k*^2^)).

**Figure 3:**
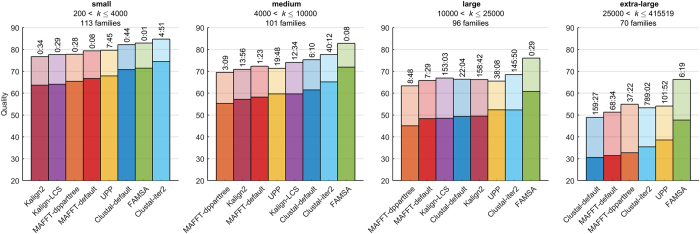
Comparison of algorithms on extHomFam. The solid bars (lower) represent TC scores, while the transparent ones (higher) —SP scores. For each subset, the algorithms were sorted in an increasing order according to the TC measure. Execution times are provided above the bars in an *hours:minutes* format.

The experiments on extHomFam confirmed superior accuracy and execution time of FAMSA to scale well with the number of sequences (Fig. 3; more detailed results are given in Supplementary material). FAMSA was inferior to Clustal-iter2 by a small margin, and only on the *small* subset. For k > 4000, it became the best aligner and, depending on the subset and quality measure, was followed by Clustal, MAFFT, or UPP. As MUSCLE and MAFFT -parttree rendered inferior results, they were excluded from Fig. 3. Kalign2, Kalign-LCS, and MUSCLE did not complete the analyzes on *extra-large* due to excessive memory or time requirements. Clustal Omega and MAFFT-default failed to process, respectively, one and four largest extHomFam families (the missing MAFFT results were taken from -dpparttree variant, though). Advances in SP and TC measures of FAMSA over the competing software on *medium*, *large*, and *extra-large* subsets were assessed statistically with the use of the Wilcoxon signed-rank test with the Bonferroni-Holm correction for multiple testing. The differences are significant at *α* = 0.005; *p*-values for all pairwise comparisons can be found in Table 2.

**Figure 4:**
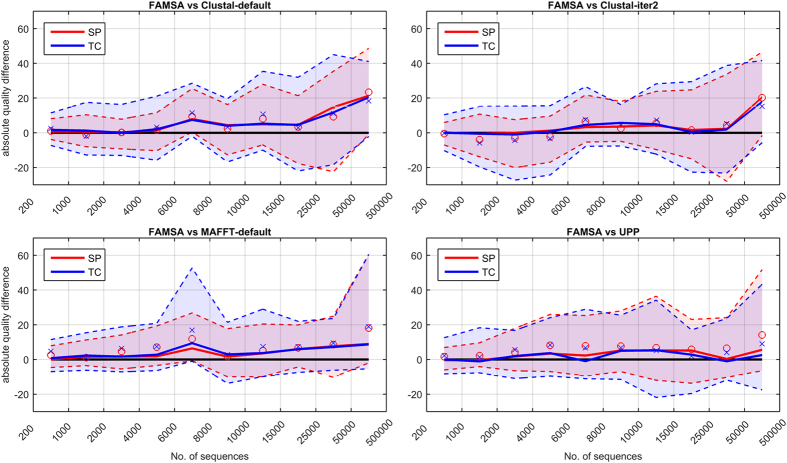
Absolute differences in SP (red) and TC (blue) scores between FAMSA and competing software for extHomFam subsets. Each interval at the horizontal axis contains approximately 35 families. Medians are represented as solid lines. Dashed lines indicate 12.5th and 87.5th percentiles (thus, filled areas contain 75% of the observations). Means are additionally given by circular (SP) and cross (TC) markers.

**Figure 5:**
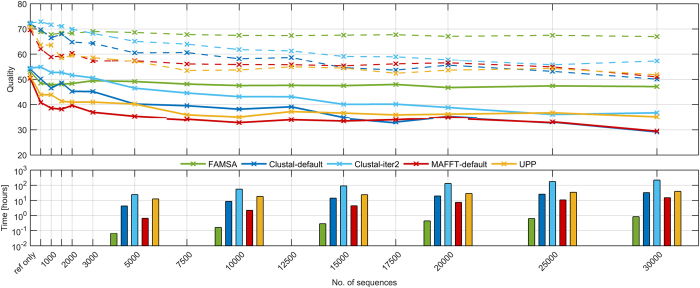
Scalability of SP (dashed lines) and TC (solid lines) scores with respect to the number of sequences. Experiments were performed on the 53 largest extHomFam families, randomly resampled to obtain the desired set size. Processing times for selected values of *k* are provided as bar plots.

As can be seen in Fig. 3, the quality advance of the presented software over other algorithms increased for consecutive subsets. For instance, on *extra-large*, FAMSA aligned in a proper manner approximately 25% more columns than UPP—the second best algorithm. A more detailed analysis of FAMSA accuracy compared to the competitors is given in Fig. 4. Four extHomFam categories were further divided into 11 subsets of approximately 35 families. Selected statistical indicators (median, mean, 12.5th and 87.5th percentile) of absolute differences in SP and TC measures between FAMSA and other algorithms were plotted for each interval at *k* axis. Clearly, the number of test cases for which the presented software was superior to that made by the competitors, as well as the absolute advance in terms of quality, increases with the growing set size. This observation is supported by the scalability analysis performed on the 53 largest families (*k* ≥ 30000), randomly resampled to obtain less numerous sets. Figure 5 shows that FAMSA outrun the competitors when the number of proteins exceeded 5000. Importantly, the performance was hardly affected when more sequences were added. This might be caused by the bias of the guide trees towards reference sequences. Indeed, the following section shows that the reference sequences were slightly closer to each other in the guide trees than suggested by the random model. Nevertheless, this held for all analyzed algorithms, therefore can be considered as a property of the benchmark.

The abundance of extremely large protein families makes extHomFam the most demanding benchmark in terms of computational resources. Apart from FAMSA, only MAFFT-dpparttree and -parttree were able to process all of its sets. Other algorithms either crashed due to memory requirements or were terminated on purpose when the processing time of a family was longer than 24 hours. (An exception was made only for Clustal-iter2 due to its superior quality results.) While MAFFT-default, MAFFT-dpparttree, UPP, and Clustal-default required from 50 to 188 hours, FAMSA finished the computations in less than 7 hours, which corresponds to 7- to 26-fold advance. Clustal-iter2 was an extreme case; it needed almost 1000 hours, and showed its combined iterations to be inapplicable for very large protein families. A more detailed analysis of the computational scalability of the presented algorithm is provided in Fig. 5. It confirms that FAMSA is faster than MAFFT-default, Clustal Omega, and UPP by 1–2 orders of magnitude. The efficiency of the presented algorithm is due to the fast bit-parallel similarity computation and the in-place profile joining. Yet, as FAMSA calculates more distances than Clustal Omega and MAFFT-dpparttree (*O*(*k*^2^) instead of *O*(*k* log *k*)), it might be expected to exceed competitor execution times for a sufficiently large *k*. To verify this, the algorithms were compared on *ABC_tran*, the largest family in extHomFam with 415 519 proteins. FAMSA processed this set in less than 2 hours. Clustal Omega crashed due to excessive memory requirements after 55 hours of calculations suggesting that the algorithm is dominated by stages other than similarity computation. The situation was different for MAFFT-dpparttree. Its execution time scaled better with the number of sequences, though it was still inferior to FAMSA by a factor of 2.5. Importantly enough, FAMSA required below 8 GB of RAM, while MAFFT-dpparttree needed 47 GB. For comparison, MAFFT-default and Clustal Omega failed to run on a 128 GB machine (the former demanded 318 GB just for storing the similarity matrix). In conclusion, the calculation of all pairwise similarities performed by FAMSA did not prevent it from being the fastest and most memory efficient aligner in the comparison–even for immense protein families.

**Figure 6:**
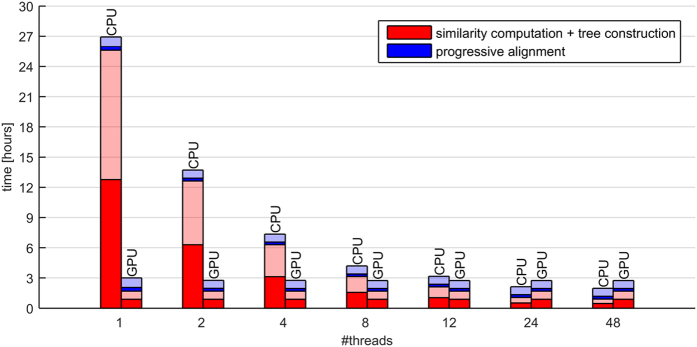
Computational scalability of FAMSA with respect to the number of cores evaluated on the ten largest extHomFam families (*k* ≥ 100000). The algorithm stages are represented by different colors. Execution times of the largest set (*ABC_tran*) are marked with solid fill, the other families are printed in with transparency.

As FAMSA was designed to use all the available computational power, it takes advantage of multi-core architectures of contemporary computers. The ten largest protein families from extHomFam (all which contain at least 100 000 sequences: *ABC_tran*, *gtp*, *HATPase_c*, *helicase_NC*, *kinase*, *mdd*, *response_reg*, *rvp*, *sdr*, *TyrKc*) were selected to investigate the scalability of the algorithm stages with respect to the number of computing threads. The experiments also considered the variant of FAMSA in which similarity calculation was adapted for massively parallel architectures with a use of OpenCL. For convenience, processing times of *ABC_tran* were marked separately. As can be seen in Fig. 6, when FAMSA was run serially, more than 90% of the execution time was related to stages I and II (the algorithm performs them simultaneously). Nevertheless, as pairwise similarities can be calculated independently, these stages scale noticeably better with the number of threads than the progressive construction. In particular, when more than 12 cores were involved, stage III of the algorithm started to be the bottleneck. This was also the case for the GPU FAMSA variant.

### Impact of guide tree computation method

A single linkage method for guide tree determination was used in FAMSA owing to low memory requirements of the SLINK algorithm and superior quality results reported in the previous studies. Nevertheless, as the tree structure was shown to be of crucial importance for the analysis of large protein families^41^, alternative methods were examined. The first one was UPGMA^27^, which can be computed in *O*(*k*^2^) time and space. The memory consumption is actually close to 2*k*^2^ bytes. This is equivalent to about 345 GB for the largest family (*ABC_tran*) making UPGMA unfeasible for immense sets of sequences. The performance of trees produced by Clustal Omega was also investigated in the experiments (FAMSA provides the user with the possibility to import external trees in the Newick format). Finally, we examined chained guide trees^41^,^42^,^43^. This method was the fastest, as it did not require calculation of sequence similarities.

The comparison of the results for extHomFam is given in Table 3. As UPGMA was non-computable for *ABC_tran*, the results of the single linkage were considered in this case. The experimental results for chained trees were averaged for 21 trials.

**Figure 7:**
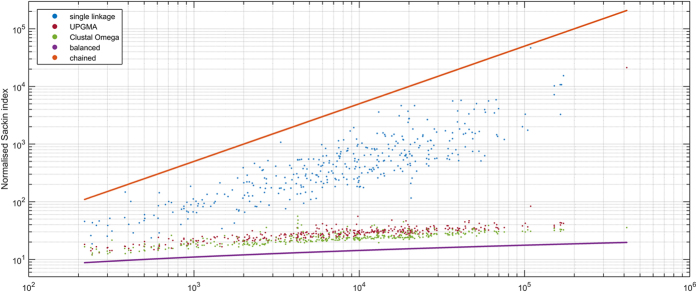
The normalized Sackin indexes (Sackin indexes divided by the cardinality of input sets) for various guide tree computation methods.

The experiments confirmed that the single linkage was superior in terms of alignment quality. The smallest, yet statistically significant advance (*p*_SP_ = 0.000011, *p*_TC_ = 0.000019), was observed when compared with UPGMA. This, together with memory efficiency, made the authors choose the single linkage for FAMSA. To provide a deeper insight into the structure of the trees rendered by different strategies, the Sackin index^44^ was used, defined as the sum of heights of all leaves in the tree. Figure 7 shows the comparison of the normalized Sackin indexes (i.e. the Sackin indexes divided by the number of sequences in the family) for trees produced by FAMSA + single linkage, FAMSA + UPGMA, and Clustal Omega. The lines corresponding to perfectly balanced and imbalanced (i.e. chained) trees are also presented for convenience. It can be seen that the indexes for single linkage trees are noticeably higher than those for UPGMA and Clustal Omega. Interestingly enough, the normalized Sackin indexes for UPGMA and Clustal Omega trees are approximately twice as large as in the perfectly balanced case.

The last experimental step involved the analysis of the guide tree structure in terms of the reference alignments coverage (Table 4). For each family the fraction of a guide tree covering reference sequences was calculated. It was defined as the number of leaves in the smallest part of the tree containing all reference sequences divided by the family size. The results were averaged over all families in the benchmark. The obtained fractions were then compared with a random model determined by performing a Monte Carlo simulation (1000 trials) for each guide tree. The trial involved a random selection of a sequence subset with the same cardinality as the reference alignment.

Interestingly enough, the fractions for the single linkage were smaller than those for UPGMA and Clustal Omega. This was probably caused by single linkage trees being noticeably more imbalanced. It can also be seen that for all tree generation algorithms, fractions containing reference sequences were smaller than the corresponding Monte Carlo results (when analyzing the entire extHomFam, the difference varied from 0.14 to 0.19). This means that the reference sequences were located in the guide trees closer to each other than suggested by the random model.

The same analysis was performed on the resampled sets employed for scalability experiments. The results coincide with the observations made for the entire benchmark.

### Classic benchmark evaluation

For completeness, the accuracy of algorithms was investigated on classic benchmarks with families ranging from a few to approximately one hundred sequences (Table 5). As expected, consistency-based methods (QuickProbs 2, MSAProbs, and GLProbs) were superior to the competitors. When it comes to the non-consistency approaches, FAMSA was characterized by moderate performance on the majority of the benchmarks, except SABmark, on which it was the best. The analysis of execution times confirms that FAMSA is one of the fastest algorithms for low and moderately-sized sets, like those contained in the investigated benchmarks.

## Discussion

The abundance of protein families containing hundreds of thousands of members imposes the development of algorithms computationally capable of aligning immense sets of sequences. The traditional progressive scheme was successfully modified by Clustal Omega and MAFFT aligners to eliminate its greatest bottleneck in large-scale analyzes—calculation of all pairwise similarities. Nevertheless, the experiments with FAMSA show that the computation of the entire similarity matrix with the use of LCS measure, combined with a memory-efficient single-linkage tree construction and in-place profile alignment, is orders of magnitude faster than the competing solutions. Importantly enough, this comes with superior alignment quality—FAMSA was significantly more accurate than Clustal Omega and MAFFT on sets of a few thousand and more sequences. *ABC_tran*, the largest among the investigated families containing 415 519 sequences, reveals the potential of the presented software. The set was processed by FAMSA within 2 hours in less than 8 GB of RAM, which is suitable for a typical laptop. In contrast, Clustal Omega crashed after 2 days of computations on a 128 GB machine due to excessive memory requirements. MAFFT in memory-efficient mode completed the analysis in 5 hours allocating 47 GB of RAM, yet only 5.7% of columns were successfully aligned, while FAMSA restored as much as 16.8%. No columns were aligned properly by UPP.

The scalability of the presented algorithm in terms of the alignment quality as well as time and memory requirements, makes it applicable for protein families even of a million sequences—a no-go area for the competing software. Such families will likely be present in the Pfam database in the near future, as a consequence of advances in sequencing technologies. Importantly enough, the efficiency of FAMSA has the potential to be further improved. The natural option is the parallelization of the dynamic programming procedure at the profile construction stage; this appeared to be a bottleneck in the scalability tests. Another possibility could be better utilization of massively parallel architectures by optimizing the OpenCL code for GPUs or adapting it for Intel Xeon Phi co-processors.

An alternative development direction concerns the alignment quality. Iterative refinement is one of the numerous techniques designed to improve accuracy. For computational reasons, it is performed by FAMSA on families of fewer than 1000 sequences, though. Some limited, less time-consuming refinement scheme could be applied also for larger sets of sequences. Other ideas include the introduction of profile Markov models or consistency. Until recently the latter was found infeasible for large families because of excessive computational requirements. However, our latest research^19^ shows that consistency applied to a small, carefully selected fraction of sequences, may improve the alignment quality without compromising on the execution time. The experiments involved sets of up to a thousand of sequences. Accordingly, the scalability of the presented ideas to families two orders of magnitude larger is an open question. Moreover, designing a consistency scheme suitable for FAMSA is by no means an easy task.

Another issue related to large-scale analyzes is an accuracy assessment, particularly the unavailability of reference sequences. Evaluating the quality of the alignment of 10 000 or more proteins on the basis of a reference containing only a small fraction of the members is the largest flaw of the experimental pipeline used in the current research. The authors believe that the progress in multiple alignment domain is likely to be facilitated with the development of new benchmark datasets containing more reference sequences.

An interesting attempt into this direction is the recent work by Fox *et al*.^45^. The proposed ContTest benchmark predicts a contact map for some protein that has a known three-dimensional structure on the ground of the evaluated multiple sequence alignment. Then the contact map is compared with the known contact map for the same protein. The benchmark contains families up to 44 thousand sequences.

FAMSA executables together with the source code are available at https://github.com/refresh-bio/FAMSA; extHomFam can be downloaded from http://dx.doi.org/10.7910/DVN/BO2SVW. Web service for remote analyzes is under development.

## Additional Information

**How to cite this article**: Deorowicz, S. *et al*. FAMSA: Fast and accurate multiple sequence alignment of huge protein families. *Sci. Rep.* **6**, 33964; doi: 10.1038/srep33964 (2016).

## Supplementary Material

Supplementary Information

## Figures and Tables

**Table 1:** Comparison of algorithms on HomFam.

Algorithm	93 ≤ *k* ≤ 3000			3000 < *k* ≤ 10000			10000 < *k* ≤ 50157			All		
	41 families			33 families			18 families			92 families		
	SP	TC	time	SP	TC	time	SP	TC	time	SP	TC	time
FAMSA	83.2	64.3	**34**	**88.3**	**71.3**	**2:36**	**79.4**	**56.9**	**8:25**	**84.3**	65.4	**11:35**
Clustal-iter2	**86.3**	**71.5**	2:14:18	85.0	68.9	14:33:00	69.5	48.3	34:50:54	82.5	**66.0**	51:38:12
Clustal-default	85.7	70.8	27:02	82.7	63.9	2:24:36	67.6	46.4	5:51:10	81.1	63.6	8:42:50
MAFFT-default	81.9	64.0	2:15	80.8	57.6	23:55	69.1	46.2	2:05:52	79.0	58.2	2:32:02
MAFFT-parttree	77.0	55.2	2:43	72.4	46.6	15:38	58.0	33.0	43:08	71.6	47.8	1:01:29
MAFFT-dpparttree	80.3	61.2	10:40	79.0	54.5	57:41	63.5	37.8	1:57:02	76.5	54.2	3:05:23
UPP	79.8	62.0	2:58:50	81.8	64.3	6:45:21	61.9	42.2	6:48:59	77.0	59.0	16:33:10
Kalign-LCS	79.8	61.3	4:17	80.6	57.6	1:31:45	67.9	44.4	64:23:14	77.8	56.7	65:59:16
Kalign2	77.4	56.2	7:04	77.6	57.1	2:30:45	64.8	41.6	97:48:46	75.0	53.7	100:26:35
MUSCLE-default	72.0	53.2	35:35:44	—	—	—	—	—	—	—	—	—
MUSCLE-maxiters2	71.8	51.4	12:35	67.1	41.6	2:27:51	40.6	21.6	30:35:03	68.8	42.1	33:15:29

**Table 2:** Statistical significance of FAMSA advances over selected competitors on extHomFam; *p*-values were calculated using the Wilcoxon signed-rank test.

Algorithm	*medium*		*large*		*extra-large*	
	SP	TC	SP	TC	SP	TC
UPP	<10^−9^	<10^−8^	<10^−7^	0.00002	<10^−6^	0.00168
Clustal-iter2	<10^−8^	<10^−7^	<10^−7^	<10^−5^	0.00001	0.00002
Clustal-default	<10^−12^	<10^−11^	<10^−8^	<10^−6^	<10^−7^	<10^−6^
MAFFT-default	<10^−16^	<10^−16^	<10^−11^	<10^−11^	<10^−9^	<10^−8^
MAFFT-dpparttree	<10^−20^	<10^−20^	<10^−14^	<10^−13^	<10^−11^	<10^−11^
Kalign-LCS	<10^−6^	<10^−7^	<10^−13^	<10^−12^	—	—

**Table 3:** Comparison of guide tree construction algorithms on extHomFam.

Algorithm	*small*			*medium*			*large*			*extra-large*					
	200 < *k* ≤ 4000			4000 < *k* ≤ 10000			10000 < *k* < ≤ 25000			25000 < *k* ≤ 415519			All		
	113 families			101 families			96 families			70 families			380 families		
	SP	TC	time	SP	TC	time	SP	TC	time	SP	TC	time	SP	TC	time
FAMSA + single linkage	82.9	71.5	1:31	**82.8**	**71.9**	8:14	**76.1**	**60.8**	29:50	**66.3**	**47.7**	6:19:10	**78.1**	**64.5**	6:58:45
FAMSA + UPGMA	**83.6**	**72.6**	1:09	80.8	69.0	6:50	73.6	56.8	28:29	64.2	45.6	6:42:14	76.8	62.7	7:18:42
FAMSA + chained	77.4	66.1	2:50	74.6	62.1	17:16	64.6	48.7	45:25	53.6	35.4	3:58:40	69.0	55.0	5:04:11
FAMSA + Clustal Omega trees	79.9	67.5	—	78.9	66.1	—	72.0	55.3	—	60.0	38.8	—	74.0	58.8	—

Times are provided in the hours:minutes:seconds format. The largest family (*ABC_tran*) was analyzed using the single linkage instead of UPGMA due to memory requirements.

**Table 4:** The structure of the guide trees generated by different algorithms in terms of the coverage of reference alignments.

Dataset	single linkage			UPGMA			Clustal Omega		
	Sackin idx	Ref. frac.	MC frac.	Sackin idx	Ref. frac.	MC frac.	Sackin idx	Ref. frac.	MC frac.
*small*	147.09	0.624	0.835	21.30	0.691	0.879	18.71	0.726	0.882
*medium*	457.50	0.627	0.837	28.63	0.714	0.883	24.76	0.726	0.893
*large*	941.30	0.681	0.842	31.13	0.675	0.885	25.90	0.772	0.895
*extra-large*	3,440.74	0.750	0.857	36.09	0.697	0.901	29.39	0.839	0.910
All	—	0.662	0.841	—	0.694	0.886	—	0.758	0.893

Column “Sackin idx” presents the normalized Sackin indexes. Column “Ref. frac.” provides the fractions of guide trees covering all reference sequences. Column “MC frac.” Shows the fractions of guide trees covering randomly selected subsets of sequences, averaged over 1000 trials.

**Table 5:** Comparison of algorithms for small datasets.

Algorithm	BAliBASE			PREFAB		OXBench-X			SABmark		
	SP	TC	time	SP/TC	time	SP	TC	time	SP	TC	time
QuickProbs 2	88.0	61.7	23:41	74.2	1:41:26	89.5	80.3	1:35:35	61.1	40.8	24
MSAProbs	87.8	60.8	35:29	73.7	2:26:39	89.1	80.0	2:42:09	60.2	40.0	29
GLProbs	87.9	59.3	23:21	72.4	1:25:40	89.1	80.0	1:10:08	61.4	41.4	3:55
MAFFT auto	86.5	58.7	10:20	72.6	17:28	88.7	79.4	7:13	57.3	36.8	1:01
Clustal-iter2	84.8	56.7	67:32	71.0	2:35:46	88.5	79.5	45:30	55.2	35.7	2:52
Clustal-default	84.2	55.9	7:41	70.0	21:56	87.8	78.1	7:34	55.0	35.5	3:52
UPP	83.0	54.2	1:23:33	69.1	5:00:29	88.9	80.0	2:49:29	52.9	33.1	18:36
FAMSA	82.6	50.1	2:40	68.3	5:39	87.4	77.5	1:30	56.4	36.8	23
Kalign-LCS	83.0	50.4	29	65.9	1:51	86.8	76.4	36	55.6	35.6	2
Muscle	81.9	47.8	14:10	67.7	35:04	87.5	77.6	26:44	54.5	33.5	45
MAFFT default	81.7	47.5	1:48	68.0	5:58	86.6	76.2	1:39	53.2	33.0	39
Kalign2	81.1	47.1	37	65.5	2:03	86.3	75.9	48	52.4	32.6	2
