# Supplementary material for: FAMSA: Fast and accurate multiple sequence alignment of huge protein families
Source: Sci Rep. 2016 Sep 27;6:33964. doi: 10.1038/srep33964 (PMC5037421; doi:10.1038/srep33964)
Supplement: Supplementary Information [file srep33964-s1.pdf]

FAMSA: Fast and accurate multiple sequence alignment of huge protein families

Supplementary material

Sebastian Deorowicz Agnieszka Debudaj-Grabysz Adam Gudyś

1 Examined programs

The following programs were used in the experimental part. The running parameters are also given.

- Clustal Omega v. 1.2.0
  - `-i <input> -o <output> --threads=8`
  - `-i <input> -o <output> --threads=8 --iter=2`
- FAMSA v. 1.0
  - `-t 8 <input> <output>`
- GLProbs v. 1.0
  - `-num_threads <input> -o <output>`
- Kalign v. 2.04
  - `-quiet -i <input> -o <output>`
- Kalign-LCS v. 2.04
  - `-quiet -b upgma -d lcs_indel -i <input> -o <output>`
- MAFFT v. 7.221
  - auto: `--auto --quiet --thread 8 --anysymbol <input>`
    - * In this mode MAFFT applies consistency-based method for 212 of 218 files of BaliBASE and for 312 of 395 files of OXBench-X. The other files in BaliBASE and OXBench-X are processed using FFT-NS-i method. All files in PREFAB and SABmark are processed using consistency-based method.
  - default: `--quiet --thread 8 --anysymbol <input>`
  - parttree: `--quiet --thread 8 --anysymbol --parttree <input>`
  - dpparttree: `--quiet --thread 8 --anysymbol --dpparttree <input>`
- MSAProbs v. 0.9.7
  - `-num_threads <input> -o <output>`
- MUSCLE v. 3.8.31
  - default: `-quiet -in <input> -out <output>`
  - maxiters2: `-quiet -in <input> -out <output>, -maxiters 2`
- QuickProbs v. 2

  - `-t 8 <input> -o <output>`

- UPP v. 1.1
  - `-m amino -s <input> -o <output> -x 8 -p ./upp-temporary`

2 Additional results

Table 1: Comparison of algorithms for ExtHomFam datasets. Times are given in hours:minutes:seconds format.

Algorithm	Small $200 < k \leq 4000$ 113 families			Medium $4000 < k \leq 10000$ 101 families			Large $10000 < k <\leq 25000$ 96 families			Extra large $25000 < k \leq 415519$ 70 families			All 380 families		
	SP	TC	time	SP	TC	time	SP	TC	time	SP	TC	time	SP	TC	time
FAMSA	82.9	71.5	1:31	**82.8**	**71.9**	8:14	**76.1**	**60.8**	29:50	**66.3**	**47.7**	6:19:10	**78.1**	**64.5**	6:58:45
Clustal Omega –iter2	**84.7**	**74.4**	4:51:52	77.7	65.2	40:12:09	68.5	52.4	145:50:07	53.3	35.5	789:02:35	73.0	59.2	979:56:45
Clustal Omega	82.2	70.8	44:39	75.3	61.5	6:10:59	66.4	49.4	22:04:32	48.9	30.6	159:27:36	70.2	55.5	188:27:47
Kalign-LCS	77.7	64.1	29:00	74.1	59.6	12:34:24	66.9	48.5	153:03:14	—	—	—	—	—	—
Kalign2	76.8	63.7	34:41	70.9	57.2	13:56:43	66.2	49.5	158:42:07	—	—	—	—	—	—
MUSCLE maxiters2	70.8	57.1	53:38	55.0	38.6	11:23:48	40.0	25.7	80:18:23	—	—	—	—	—	—
MAFFT default	79.4	66.8	8:38	72.4	58.2	1:23:43	65.8	48.3	7:29:33	51.3	31.5	68:34:47	68.9	53.3	77:36:43
MAFFT parttree	74.8	61.7	8:07	64.6	48.3	51:48	54.9	38.0	3:16:10	45.7	24.5	25:48:33	61.7	45.3	30:04:40
MAFFT dpparttree	77.8	65.5	28:54	69.5	55.3	3:09:57	63.4	45.1	8:48:17	54.9	32.8	37:22:16	67.7	51.6	49:49:26
UPP	79.7	67.9	7:45:53	71.3	59.7	19:47:59	65.5	52.4	38:08:16	54.1	38.6	101:52:53	67.4	56.4	167:35:01
